# Supplementary material for: Trans-Cinnamaldehyde Attenuates Enterococcus faecalis Virulence and Inhibits Biofilm Formation
Source: Antibiotics (Basel). 2021 Jun 11;10(6):702. doi: 10.3390/antibiotics10060702 (PMC8230787; doi:10.3390/antibiotics10060702)
Supplement: Supplementary file 1 [file antibiotics-10-00702-s001.zip › antibiotics-1253461-supplementary.pdf]

antibiotics

MDPI

Article

Trans-Cinnamaldehyde Attenuates *Enterococcus faecalis* Virulence and Inhibits Biofilm Formation

Islam A. A. Ali [1], Jukka P. Matinlinna [1], Celine M. Lévesque [2] and Prasanna Neelakantan [1,*]

[1] Faculty of Dentistry, The University of Hong Kong, Hong Kong, China; u3004980@connect.hku.hk (I.A.A.A.); jpmat@hku.hk (J.P.M.); prasanna@hku.hk (P.N.)
[2] Faculty of Dentistry, University of Toronto, Toronto, ON M5G 1G6, Canada; Celine.Levesque@dentistry.utoronto.ca
* Correspondence: prasanna@hku.hk; Tel.: +852-2859-0581

Table S1. Sequence of primers used in the qRT-PCR analysis.

Gene	Sequence (5'- 3')	Function	References
23S rRNA	F- CCTATCGGCCTCGGCTTAG<br>R- AGCGAAAGACAGGTGAGAATCC	Housekeeping gene	[1]
fsrB	F- TGCTCAAAAAGCAAAGCCTTATAA<br>R- GATGACGAGACCGTAGAGTATTACTGAA	Processing of a peptide precursor to generate GBAP *	[1]
fsrC	F- GCTTATTTGGAAGAACAACGTATCAA<br>R- CGAAACATCGCTAGCTCTTCGT	Histidine kinase sensor of the Fsr quorum sensing system	[1]
gelE	F- CGGAACATACTGCCGGTTTAGA<br>R- TGGATTAGATGCACCCGAAAT	Production of an extracellular protease involved in biofilm formation and degradation of host tissues	[1]
luxS	F- TGCAGAGACTGCTGGAACAACTT<br>R- GGGTTGCCGCACTGGTTTTTATATGA	Encodes a metalloenzyme involved in AI-2 production	This study
ftsZ	F- CCGTCAAACAAGACAAGCGG<br>R- TCCCAATCGCCAAAAGCACT	Cell division	[2]

* GBAP: Gelatinase Biosynthesis Activating Pheromone, the peptide autoinducer of the Fsr-quorum sensing system in *E. faecalis*.

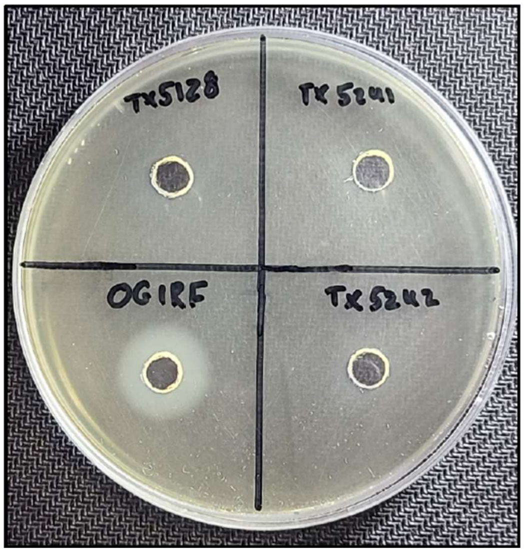

Figure S1. Extracellular protease activity of the wild-type OG1RF, *gelE* (TX 5128), *fsrB* (TX 5241) and. *fsrC* (TX 5242) mutant strains. The presence of opaque zone surrounding the OG1RF strain indicates positive protease activity, while no opaque zones were observed surrounding the *fsr* or *gelE* mutants, which indicate negative protease activity.

References

1. Shepard, B.D.; Gilmore, M.S. Differential Expression of Virulence-Related Genes in Enterococcus faecalis in Response to Biological Cues in Serum and Urine. *Infect. Immun.* **2002**, *70*, 4344–4352, doi:10.1128/iai.70.8.4344-4352.2002.
2. He, Z.; Liang, J.; Zhou, W.; Xie, Q.; Tang, Z.; Ma, R.; Huang, Z. Effect of the quorum-sensingluxSgene on biofilm formation byEnterococcus faecalis. *Eur. J. Oral Sci.* **2016**, *124*, 234–240, doi:10.1111/eos.12269.
